# Detection techniques for tomato diseases under non-stationary climatic conditions

**DOI:** 10.3389/fpls.2025.1704663

**Published:** 2025-12-05

**Authors:** Zhenzhen Wu, Jiao Han, Shiyu Wang, Xiangwei Meng, Rui Fu

**Affiliations:** Shandong Facility Horticulture Bioengineering Research Center, Weifang University of Science and Technology, Weifang, Shandong, China

**Keywords:** tomato disease detection, non-stationary environments, test-time domain adaptation, knowledge distillation, dynamic data augmentation, teacher–student, pseudo-labeling, EMA

## Abstract

Tomato growth is highly susceptible to diseases, making accurate identification crucial for timely intervention. While deep learning models like the YOLO family have demonstrated success in detecting diseases in agricultural settings, they typically assume that training and testing data are independently and identically distributed (i.i.d.), which often doesn’t hold in real-world scenarios. When pre-trained models are applied to new environments, performance can degrade due to domain shifts. To address this, we propose CTTA-DisDet, a continuous test-time domain adaptation framework for tomato disease detection that adapts models to evolving environments during testing, improving generalization in unseen domains. CTTA-DisDet utilizes a teacher-student architecture where both models share the same structure. Dynamic data augmentation is introduced, involving explicit and implicit augmentations. Explicit augmentation corrupts input images, while implicit augmentation uses large language models (LLMs) to generate new domain data. The teacher model learns generalized knowledge, and the student model mimics the teacher to distill domain-specific information. During testing, pseudo-labels generated by the teacher update the student model. To prevent catastrophic forgetting, a subset of neurons is randomly restored to their original weights during each test-time iteration. The teacher model is continuously updated via exponential moving average (EMA). Experimental results demonstrate that CTTA-DisDet achieves an impressive 67.9% performance in continuously changing cross-domain environments, significantly benefiting practical applications in non-stationary settings.

## 1 Introduction

The escalating global population and climate shifts pose formidable hurdles for crop cultivation. Plant diseases, once contracted, can swiftly propagate and inflict heavy economic losses. Tomatoes, widely cultivated across temperate and tropical regions, are a vital food crop due to their high nutritional and economic value. However, like many crops, they are vulnerable to various diseases during growth (Benelli et al., 2020), which can drastically reduce yields if not detected in time (Liu and Wang, 2020).

Traditional early detection often relies on manual inspection by experts, which is time-consuming and error-prone. In recent years, machine learning and computer vision have emerged as effective tools for identifying plant diseases through image analysis, offering a faster and more accurate alternative.

Several studies have explored these approaches. For instance, Lu et al. (2023) combined spectral and image data to detect cotton yellow wilt disease, while Zhou et al. (2024) and Duan et al. (2024) proposed image-text retrieval and hyperspectral learning frameworks for rice and chili diseases. Other efforts, such as Triki et al. (2023) and Gao et al. (2023), applied simulation and remote sensing technologies to improve recognition performance. Although these works achieved notable progress, they primarily capture basic visual traits and still struggle to represent complex, fine-grained disease patterns, limiting their practical applicability.

In computer vision, deep learning is transforming disease detection, as evidenced by promising findings in Khalid et al. (2023) and Johnson et al. (2021). YOLO-based detectors and other neural network architectures have demonstrated notable advantages in agricultural applications, offering both high accuracy and real-time inference (Abade et al., 2021; Zheng et al., 2022). For example, MG-YOLO (Li et al., 2023) improves spore detection in cases of blur, dense clustering, and irregular morphology, achieving a 6.8% improvement over YOLOv5. SeptoSympto (Mathieu et al., 2024) leverages a U-Net and YOLO hybrid to quantify wheat leaf necrosis, while Torres-Lomas et al. (2024) introduced an image–text paired model for tomato disease diagnosis without manual annotation. Transformer-based segmentation approaches, such as Li et al. (2024a), have further enhanced feature extraction for rice leaf disease localization. Beyond detection, several studies explore model robustness, transferability, and fine-grained segmentation. Li et al. (2024b) evaluated multiple pre-trained CNNs for plant disease diagnosis under transfer learning, while MC-UNet Deng et al. (2023) integrates multi-scale fusion for tomato disease segmentation. DC2Net (Feng et al., 2024) incorporates deformable and dilated convolutions for Asian soybean rust detection, setting new performance benchmarks. Weakly supervised and few-shot segmentation has also been explored in Zhou et al. (2023), while Tang et al. (2023) employed proximity feature aggregation to handle inter- class similarity in custom datasets. Additionally, Dong et al. (2023) released a comprehensive set of pre-trained models for plant disease recognition, facilitating downstream applications. Temporal monitoring strategies such as Tschurr et al. (2023) utilized high-resolution time-series imagery for canopy frost assessment, and Xu et al. (2023) demonstrated the effectiveness of hyperspectral and RGB-based deep learning models for tea coal smut classification, outperforming traditional baselines such as SVM and showcasing the potential of multimodal learning.

Especially, You Only Look Once (YOLO) is an advanced real-time object detection system. It processes images at 30 FPS on a Pascal Titan X and achieves a mean average precision (mAP) of 57.9% on the COCO test-dev dataset. Recent research closely related to crop diseases includes several advancements (Zhu et al., 2023; Qi et al., 2023; Dong et al., 2024. Dai et al. (2023) enhanced the YOLOv5m model by incorporating Swin Transformer (SWinTR) and Transformer (C3TR) mechanisms for plant pest detection. Tests showed 95.7% accuracy, 93.1% recall, an f2 score of 94.38%, and an mAP of 96.4%, outperforming the original YOLOv3, YOLOv4, and YOLOv5m models. Ye et al. (2023) enhanced the YOLOv5s algorithm for detecting pine wilt disease (PWD), creating the PWD-YOLO model. It offers superior efficiency with a compact size of 2.7 MB, 3.5 GFLOPs complexity, 1.09 MB parameters, and processes 98.0 frames/s on their tailored dataset.

**Figure 1:**
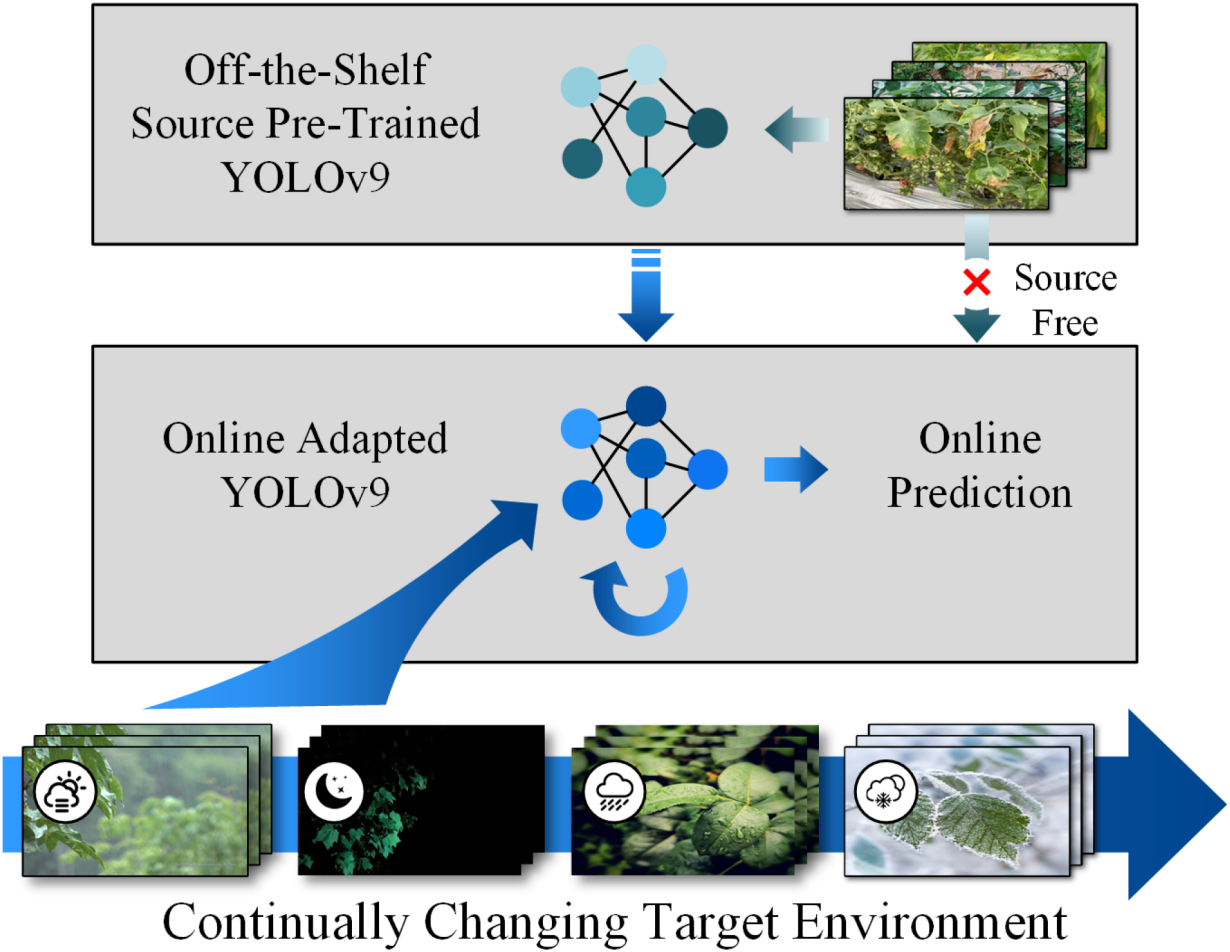
In dynamic real-world settings (e.g., varying weather), our target model starts with a pre-trained source network. It updates in real-time using current data without needing source data, overcoming issues like error buildup and forgetting to maintain performance over time. Our approach ensures sustained adaptation in fluctuating environments.

The prevailing research in disease recognition tends to concentrate on static scenarios, relying on image-based identification under controlled settings. This method, albeit beneficial, is fundamentally constrained by the i.i.d. data assumption. As illustrated in **Figure 1**, such static modeling fails to maintain accuracy under real-world dynamic agricultural environments, motivating the need for adaptive frameworks like CTTA-DisDet.

Agricultural dynamics are strongly shaped by environmental fluctuations and disease progression, which cause data distribution shifts that degrade model accuracy. Variability in symptom appearance under different weather conditions often leads to misclassification when models are trained on static datasets. For example, an atypical blight strain in a tomato plantation may be overlooked by a non-adaptive model, delaying treatment and increasing losses. Frameworks like CTTA-DisDet are designed to remain adaptive at inference time, thereby supporting timely and reliable diagnosis in real-world agricultural environments.

As machine learning evolves, researchers have increasingly focused on addressing such distribution gaps through test-time adaptation (TTA), which dynamically refines the model on the target domain without requiring access to source data (Chi et al., 2021; Varsavsky et al., 2020; Sun et al., 2020; Wang et al., 2020). Recent studies have validated the effectiveness of TTA across multiple vision tasks: Shanmugam et al. (2021) and Xu et al. (2022) show that improved aggregation strategies during testing can significantly enhance classification robustness, while Cohen et al. (2023) demonstrates that diverse unsupervised augmentations reduce false positives in anomaly detection. In agriculture, Saadati et al. (2024) applied TTA to pest classification and incorporated OOD awareness to avoid unreliable predictions on unfamiliar species, and Cui et al. (2023) proposed a teacher–student TTA scheme based on domain-augmented training to improve cross-domain generalization.

In this work, to handle the challenges of tomato disease detection in non-static scenarios, we draw inspiration from the recent progress in TTA and knowledge distillation. We further take into account the dynamic nature of real-world tomato plantations, where environmental factors such as weather, pest outbreaks, and disease progression constantly introduce domain shifts, causing each target-domain sample to exhibit different visual characteristics.

To address this issue, we propose a novel continuous test-time adaptation framework, CTTA-DisDet, which enables the model to adapt online during inference, thereby improving generalization in unseen environments. Specifically, CTTA-DisDet adopts a teacher–student design with identical network architectures. Both models are based on YOLOv9, which provides a favorable balance between detection accuracy and computational cost in practical deployment; meanwhile, the framework itself is model-agnostic and can be readily extended to other detectors such as YOLOv10, Fast R-CNN, or SSD.

Unlike the student model, which directly processes the original images, the teacher model is trained on data generated by our Dynamic Data Augmentation (DDA) pipeline. Following recent advances in knowledge distillation, DDA incorporates both explicit and implicit augmentations: the explicit augmenter generates *M* = *K* × *S* corruption-based variants per image using *K* transformation strategies and *S* fixed perturbation levels, while the implicit augmenter leverages multimodal LLMs [e.g., GPT4o (Islam and Moushi, 2024)] to synthesize additional domain-diverse samples guided by textual prompts. A fidelity classifier further filters inconsistent or irrelevant samples to ensure augmentation quality.

Notably, by combining fixed explicit perturbations with flexible LLM-driven implicit augmentations, CTTA-DisDet benefits from both controlled domain expansion and realistic distribution shifts, enabling effective test-time adaptation and improved robustness in previously unseen agricultural environments.

In summary, this work introduces CTTA-DisDet, a state-of-the-art solution for tomato disease detection under non-stationary conditions, with the following key contributions:

- We propose a continuous test-time domain adaptation framework (CTTA-DisDet), which allows the integration with the mainstream YOLO detection models. This flexibility ensures that CTTA-DisDet can be accommodated different levels of computational resources and performance.
- CTTA-DisDet features a dynamic data augmentation (DDA) that encompasses explicit and implicit augmenters. The former create varied image corruptions to simulate environmental perturbations, while the latter use LLMs to synthesize new domain-relevant data. This comprehensive augmentation enriches the model’s training, enhancing its adaptability to diverse and changing conditions.
- By employing a teacher-student configuration, CTTA-DisDet leverages knowledge distillation to transfer cross-domain generalization capabilities. The teacher model, trained on augmented data, imparts its learned knowledge to the student model, which is then fine-tuned for the specific target domain, ensuring improved accuracy in detecting diseases across different environmental contexts.
- Through rigorous experimental validation, we demonstrate that CTTA-DisDet achieves an impressive 67.9% detection accuracy in continuously changing cross-domain environments, outperforming conventional models. This significant enhancement in performance not only underscores the framework’s technical superiority but also highlights its practical applicability, offering a concrete advantage for agricultural stakeholders by facilitating more precise and timely disease detection and management.

## 2 Materials and methods

### 2.1 Overview

**Figure 2:**
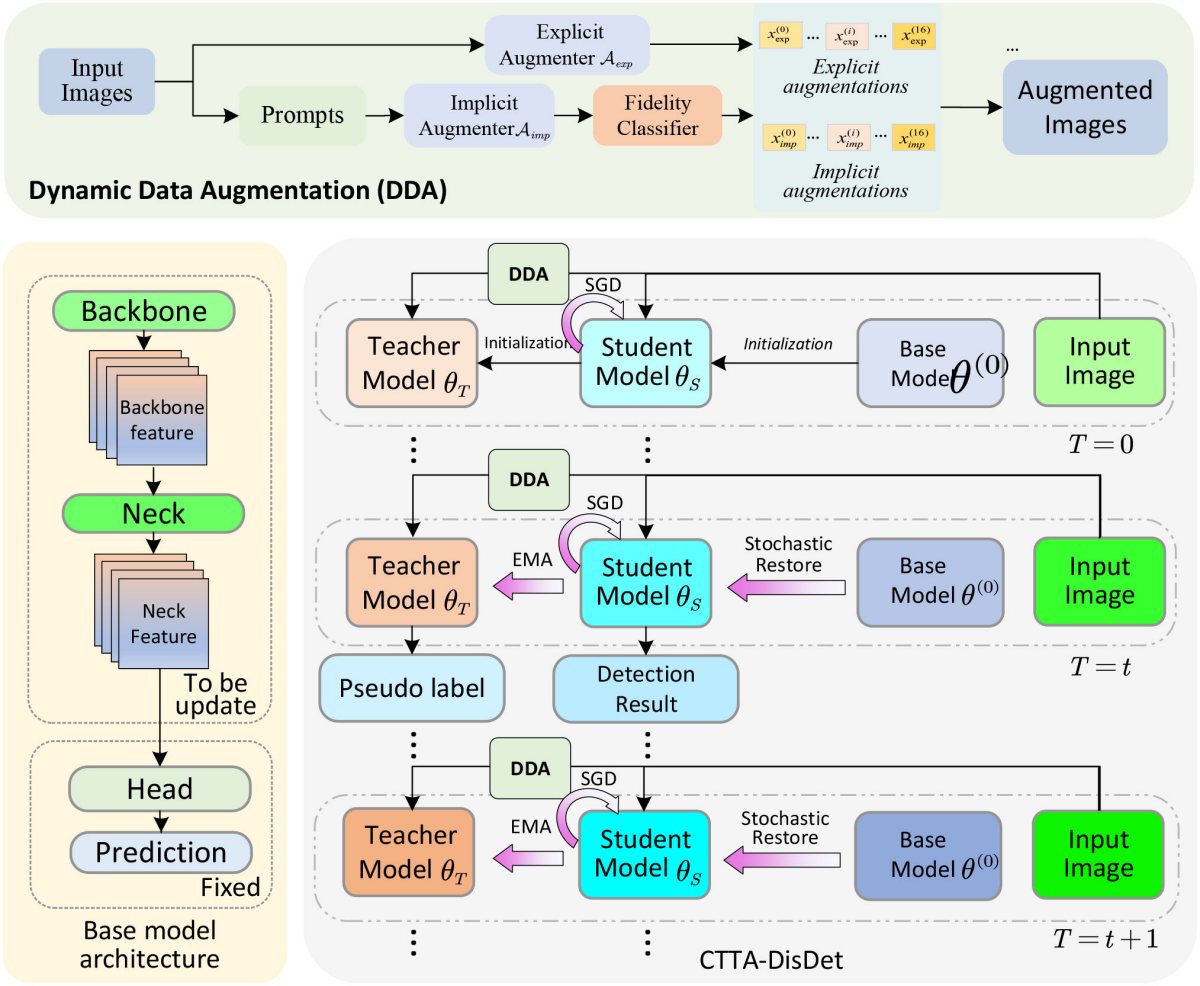
Illustration of the proposed continuous test-time domain adaptation framework (CTTA- DisDet). The backbone follows the network structure of the YOLOv9 model; however, includes a knowledge distillation architecture with a teacher model *θ_T_* and a student model *θ_S_*. Moreover, the dynamic data augmentation (DDA) generates augmented images that adapt to changing environmental conditions, thereby enhancing the model’s generalization ability in unseen domains. We note the training phase of CTTA-DisDet is similar to the conventional training process; by contrast, at test-time, the model continuously adapts to the changing environment, improving its performance in non-stationary scenarios. This continual adaptation is achieved through the teacher-student model architecture, where the teacher model *θ_T_* learns generalized knowledge from augmented images, and the student model *θ_S_* distills domain-specific information.

To achieve the goal of tomato disease detection in non-stationary environments, we propose a novel continuous test-time domain adaptation framework (CTTA-DisDet) that combines the advantages of both knowledge distillation and dynamic data augmentation. Compared with the vanilla YOLO, our proposed CTTA-DisDet consists of two innovative components: dynamic data augmentation (DDA) and continuous test-time domain adaptation (CTTA). The proposed CTTA-DisDet framework is illustrated in **Figure 2**.

In this framework, the teacher and student models are constructed based on the same YOLOv9-s backbone and neck to ensure architectural consistency. No additional attention modules are introduced, and parameters are fully shared across the feature-extraction layers, while the detection heads are updated independently during the adaptation process. This design allows the teacher to provide stable supervision through EMA updates and the student to adapt flexibly to domain shifts in real time.

Current advancements have evidenced that data augmentation is a powerful technique for enhancing the generalization ability of deep learning models. However, the existing data augmentation strategies are static and do not adapt to changing environments. In this work, we propose a dynamic data augmentation (DDA) that combines both explicit and implicit augmentations $(A_{exp},A_{imp})\;$ and can dynamically generates augmented images based on the current environment conditions. $A_{exp}\;$ follows the standard data augmentation pipeline and produces 16 augmentations of input images using 4 corruption strategy (Gaussian noise, Brightness adjustment, Pixel loss and Blur noise) with 4 fixed levels. $A_{imp}$ is derived from the current cutting-edge text- to-image generative model [e.g., GPT4o (Islam and Moushi, 2024)] to better generate images with more expressiveness and diversity from text descriptions and the original image. In our work, we take 4 different environmental conditions (Foggy day, Dark night, Rainy day, Snowy day) and 4 different levels to construct the textual prompts, and generate 32 stylized images. Meanwhile, the implicit augmenter are followed by a binary classifier to filter out the generated images that are not relevant to the original image, and the remaining 16 images are used to train the implicit augmenter until sufficient stylized images are produced.

In addition to DDA, the proposed CTTA-DisDet also employs a teach-student model architecture to facilitate knowledge distillation, where the teacher $\theta_{T}$ and the student model $\theta_{S}$ share the same network architecture derived from the YOLO v9 model $\theta^{\left(0\right)}$ (Wang and Liao, 2024). Fed into the augmented images generated by DDA, $\theta_{T}$ is trained to learn the generalized knowledge. Then, the student model $\theta_{S}$ is trained to mimic the teacher using the proposed continuous test-time domain adaptation (Chi et al., 2021; Varsavsky et al., 2020), thereby distilling domain-specific information. During the test phase, for *t*-th given image, the teacher model $\theta_{T}^{\left(t-1\right)}\;$ can achieve the pseudo-label, which is then used to obtained adapted student model $\theta_{S}^{\left(t\right)}\leftarrow \;\theta_{S}^{\left(t-1\right)}\;$ through an additional gradient descent step. Moreover, to prevent catastrophic forgetting, a small subset of neurons from the pre-trained model is randomly restored to the original pre-trained weights $\theta^{\left(0\right)}$ in each test-time iteration to student model. This random restoration mechanism ensures that both teacher and student model $\theta_{S}^{\left(t\right)}\;$ can retain the original knowledge while adapting to the new domain. After the adaptation of student model, the adapted student model $\theta_{S}^{\left(t\right)}$ can produce the final prediction via a single forward pass. Then, the updated teacher model $\theta_{S}^{\left(t\right)}$ can be correspondingly achieved using the exponential moving average (EMA) (Klinker, 2011) from the previous parameter of teacher $\;\theta_{S}^{\left(t-1\right)}\;$ and the adapted student model’s parameter $\theta_{S}^{\left(t\right)}$.

### 2.2 Dynamic data augmentation

**Figure 3:**
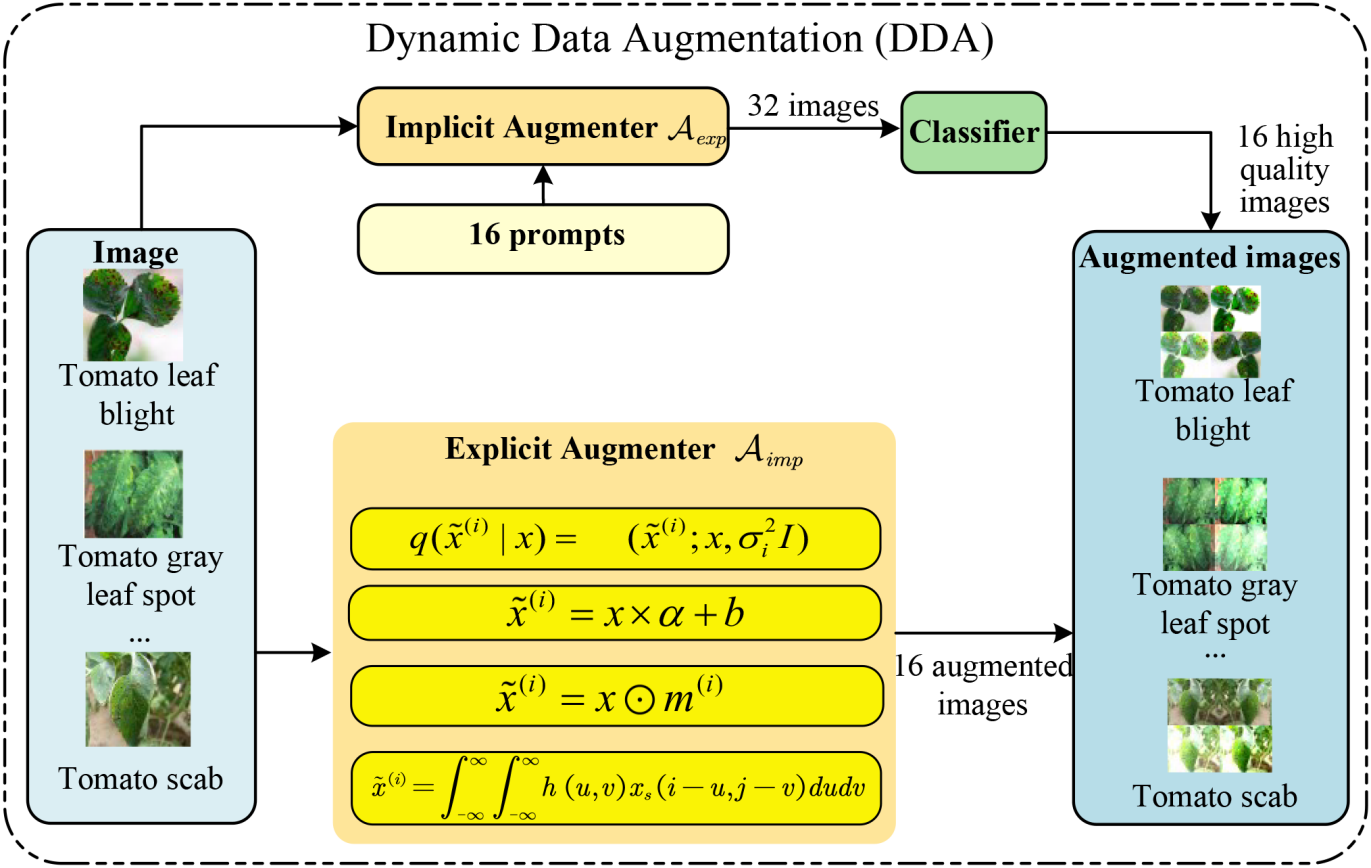
The proposed dynamic data augmentation (DDA), which involves both explicit and implicit augmenter $(A_{exp},A_{imp}).$ The former generates corrupted counterparts of input images, while the latter leverages large language models (LLMs) to produce new domain data. Then, the augmented images are fed into the teacher model to learn cross-domain generalized knowledge, and the student model is subsequently trained to mimic the teacher, thereby distilling domain-specific information, to improve the model’s generalization ability in unseen domains.

Recent advancements have demonstrated that data augmentation is a powerful technique for enhancing the generalization ability of deep learning models (Zhang et al., 2022; Fleuret, 2021; Chen et al., 2023; Wang et al., 2022). Inspired by the success of data augmentation, we propose a dynamic data augmentation (DDA) that can adapt to changing environments. DDA is designed to generate augmented images that can adapt to the changing environment conditions. The DDA consists of both explicit and implicit augmenter $(A_{exp},A_{imp}),$ which are designed to enrich the training data and distill the cross-domain generalized information, as shown in **Figure 3**. Next, we will introduce the explicit and implicit augmenters in detail.

Explicit Augmenter $A_{exp}$ enhances model generalization through various image transformations, including geometric transformations, brightness adjustments, noise additions and pixel-level transformations, which have been proven effective in numerous related literatures. We note that $A_{exp}$ is parameter-free, and the explicit augmenter is designed to generate 16 augmented images for each input image. Specifically, we select 4 common explicit augmentation methods: Gaussian noise, Brightness adjustment, Pixel loss, and Motion blur, each with 4 different parameter settings to generate a more diverse set of samples. The explicit augmenter is designed to simulate the common image variations in real-world scenarios and improve the model’s robustness in practical applications. These four explicit augmentation methods were selected because they cover common image variation scenarios and are easy to implement and control.

They have characteristics like intuitive effectiveness, easy implementation, being able to introduce uncertainty and increasing difficulty, which can effectively enrich the data samples and prompt the model to learn more comprehensive and robust feature representations, thereby improving the accuracy and generalization ability of pest and disease recognition and detection. The 4 mathematical models of the explicit augmenter are as follows:

(1) Gaussian Noise. To emulate sensor imperfections and environmental interference, Gaussian noise is added to each pixel value of the input image. This augmentation helps the model learn invariant representations and suppress overfitting to clean data. For a given image ***x***, the augmented versions ***x***^(*i*)^ are generated by the Equation 1:

(1) $$q(x^{\left(i\right)}|x)=N(x^{\left(i\right)};x,\;\sigma_{i}^{2}I),$$

where the notation $N(x^{\left(i\right)};x,\;\sigma_{i}^{2}I)$ signifies a Gaussian distribution centered at the original image ***x*** with a covariance matrix defined by the variance $\sigma_{i}^{2}$ times the identity matrix ***I***. The augmented image ***x***^(^*^i^*^)^ is thus influenced by the Gaussian noise with a standard deviation of $\sigma_{i}$. In our experiments, we selected four distinct standard deviations: *σ* = [5, 10, 15, 20] to explore the effect of varying noise levels on model performance.

(2) Brightness Adjustment. Brightness variations due to diverse lighting conditions can impede object recognition in images. To mitigate this, we apply brightness adjustment to simulate real- world lighting and improve model adaptability. The process adjusts the image intensity by scaling the original image ***x*** with a factor *α* and adding a bias ***b***, as defined in Equation 2:

(2) $$x^{\left(i\right)}=x\;\times \;\alpha +b$$

The scaling factor *α*, uniformly distributed between 0.8 and 1.2, modulates image brightness, while the bias term ***b***, ranging from -0.5 to 0.5, fine-tunes color balance, collectively enhancing model performance across varying illumination.

(3) Pixel Loss. In dynamic environments, occlusions among leaves lead to information loss, which we emulate through pixel loss augmentation. This technique involves setting random pixels to zero or replacing them with the average of neighboring pixels, mimicking real-world occlusions and prompting the model to concentrate on salient features. Let ***m***^(^*^i^*^)^ ∈ {0, 1} denote a binary mask with a uniform loss rate of *α*, the corrupted image ***x*** is generated by element-wise multiplication, as formalized in Equation 3:

(3) $$x^{\left(i\right)}\;=\;x\;⊙\;m^{\left(i\right)},$$

where ⊙ denotes the Hadamard product. The binary mask $m^{\left(i\right)}\;$ is generated by setting each pixel to zero with probability $\alpha_{i}$ and one otherwise. We set α = [0.1, 0.15, 0.2, 0.25] to create varying levels of pixel loss, enabling the model to learn robust features in the presence of occlusions.

(4) Motion Blur. This augmentation technique introduces a level of blur that mimics the effect of relative motion between the camera and the subject. Our augmenter employs a range of kernel sizes to generate varying degrees of blur: small kernels (1 × 1, 3 × 3) for a subtle effect, medium kernels (5 × 5, 7 × 7) for moderate blurring, and large kernels (9 × 9 to 15 × 15) for a pronounced blur effect. This approach enables the model to learn from a diverse set of scenarios, improving its generalization capabilities. The mathematical formulation of motion blur is encapsulated in the convolution integral in Equation 4:

(4) $$x^{\left(i\right)}=\int_{-\infty }^{\infty }\int_{-\infty }^{\infty }h(u,v)x(i-u,j-v)dudv.$$

Here, *h*(*u, v*) denotes the point spread function (PSF), which characterizes the blur induced by motion. ***x***^(^*^i^*^)^ is the resulting intensity at pixel coordinates (*i, j*), and ***x***(*i* − *u, j* − *v*) represents the original image’s grayscale values at the shifted coordinates. By analyzing *h*(*u, v*), we can understand the impact of motion blur and potentially apply de-blurring techniques to restore image clarity.

After the parameter-free explicit augmenter $A_{exp}$ with 4 corruption strategies and 4 fixed levels, we generate 16 augmented images for each input image. For a clear distinction with the implicit augmenter $A_{imp}$, we afterhere denote the explicit augmented images as $\{x_{exp}^{\left(i\right)}{\}\;}_{i=1,}^{16}$ where $x^{\left(i\right)}\;$ represents the *i*-th augmentation, and the $\{x_{imp}^{\left(i\right)}{\}\;}_{i=1}^{16}\;$ as the implicit augmented images. We note that the $A_{exp}$ is effective in simulating common image variations in real-world scenarios and improving the model’s robustness in practical applications.

Implicit Augmenter $A_{imp}\;$ represents an advanced data augmentation technique that enhances model generalization by introducing training uncertainty. This implicit augmentation method leverages Large Language Models (LLMs) to generate images from descriptive text, significantly improving the naturalness and realism of the visual content. It surpasses the limitations of traditional tools like OpenCV, which often fall short in simulating complex physical conditions and offer limited parameter flexibility. By integrating LLMs, we create a diverse set of cross-domain augmented images that reflect various weather conditions, environments, and visibility levels, leading to feature generalization prior to data input. We focus on four common environmental conditions—Foggy, Dark, Rainy, and Snowy days—each with four distinct parameter settings, totaling 16 textual prompts. These conditions are chosen for their prevalence and representativeness in real-life scenarios, as well as their potential to simulate complex weather phenomena, preparing the model for a broad range of dynamic environmental changes and enhancing its recognition efficiency in unseen domains. Each text prompt generates 2 augmented images, therefore 16 prompts for 32 implicit augmentations, which are then evaluated by a fidelity classifier that retains high-assurance images and discards those with low fidelity, retaining only the 16 higher-quality $\{A_{imp}{\}\;}_{i=1}^{16}$. This binary classifier ensures that only reasonable images are retained, avoiding the impact of extreme unreasonable results, and fortifies the model’s generalization capacity by ensuring the generated images adhere to expected features.

(1) Foggy day. To achieve diverse foggy weather effects, we utilize a textual template with four levels of fog intensity. The LLMs generate augmented images that simulate eight levels of foggy days based on the input original image. The foggy day template is as follows: Add a level fog effect to the given image, without any changes to the other context. Concretely, the four levels of fog intensity are: very slight, light, moderate, and heavy. By employing LLMs, we can create eight distinct foggy day effects, each with unique characteristics.

- Add a very slight fog effect to the given image, without any context changes.
- Add a light fog effect to the given image, without any context changes.
- Add a moderate fog effect to the given image, without any context changes.
- Add a heavy fog effect to the given image, without any context changes. ·

(2) Dark night. To imbue images with a realistic and varied nocturnal ambiance, we employ 4 prompts tailored for nighttime effects. These strategies are designed to capture the nuances of darkness that can occur in real-world scenarios. Our approach utilizes Large Language Models (LLMs) with a specific template request: “Generate different levels dark night effect to the given image”. The four levels of darkness are: slight, half, grey, and deep, as detailed below:

- Generate slight dark night effect to the given image.
- Generate half dark night effect to the given image.
- Generate grey dark night effect to the given image.
- Generate deep dark night effect to the given image.

(3) Rainy day. For this cases, the textual template is Generate different levels raining effect to the given image. The model is trained to generate 4 different levels of raining effect to the given image. The specific levels of raining effect are: drizzle, moderate, heavy, and thunderstorm, as described below:

- Generate drizzle raining effect to the given image.
- Generate moderate raining effect to the given image.
- Generate heavy raining effect to the given image.
- Generate thunderstorm raining effect to the given image.

(4) Snowy day. We utilize 4 text prompts to generate augmented images with various snowy weather effects. The template of LLMs is: Generate different levels snow effect to the given image. The four levels of snowy day are: light snow, moderate snow, heavy snow, and blizzard, as detailed below:

- Generate light snow effect to the given image.
- Generate moderate snow effect to the given image.
- Generate heavy snow effect to the given image.
- Generate blizzard snow effect to the given image.

We note that these structured textual prompts consider the diversity of weather conditions and the need for the model to adapt to various environmental changes. The model is expected to generate a variety of foggy, dark, rainy, and snowy images, each with its own unique characteristics and style. Moreover, for each prompt, we generate two augmented images, therefor, resulting in a total of *N* = 32 augmented images for 16 textual prompts.

We chose GPT-4o for its multi-modal text-to-image capability that supports fine-grained prompt control (e.g., fog density levels), unlike StyleGAN which requires domain-specific training. This capability allows flexible adaptation to agricultural scenes and ensures semantic alignment between generated and real images.

#### 2.2.1 Fidelity classifier

To filter out the generated images that are not relevant to the original image, we introduce a binary classifier to evaluate the fidelity of the generated images. The classifier is trained to distinguish between high-fidelity and low-fidelity images, ensuring that only high-quality images are retained for model training. The classifier is implemented using a pre-trained ResNet-19 model, which is fine-tuned on our dataset. After generating augmentations from LLMs, the classifier will eliminated the scenes with suboptimal effects. For example, if the generated rainy scene is overly smooth and does not conform to the expected real rainy scene characteristics, then the classifier can identify and remove such ineffective rainy scene to retain various rainy scene images that are more in line with requirements, have greater authenticity and diversity. If the generated implicit images fail to pass the classifier and there are less than 16 kinds, then a new round of image updating is needed until 32 augmented image categories are satisfied.

If we have the true label $y_{i}$ (which is a category label) and the corresponding probability distribution is $p_{i}$, while the probability distribution predicted by the model is *q*(*y*), this loss function is formally expressed in Equation 5:

(5) $$L_{fidelity}=\sum_{i=1}^{N}y_{i}\text{log}\;q_{i}+(1-y_{i}\log)(1-q_{i})$$

Among them, *N* = 32 is the number of augmented samples, *y_i_* represents the true label of the *i*th sample, and *q_i_* represents the probability value that the model predicts the sample belongs to each category (corresponding probability distribution). By minimizing this cross-entropy loss function, the prediction result of the model can be closer to the real situation. These 16 kinds of fixed explicit augmented versions together with the new domain augmented 16 kinds of images generated by the implicit augmenter, provide rich data sources for model training. In this way, the model can not only learn the generalization knowledge in the augmented domain, but also can perform personalized processing according to the characteristics of different input samples, showing higher recognition accuracy and robustness in practical applications.

### 2.3 Continuous test-time domain adaptation

In dynamic environment T, our objective is to engineer a model capable of adapting to ongoing changes for effective object detection (Cui et al., 2023; Zhang et al., 2022). To this end, we employ the target data $x_{T}^{\left(i\right)}$, where 0 *< i <* | T|, to fine-tune the base YOLO model, denoted as $\theta^{\left(0\right)}$, using our CTTA-DisDet. Our framework enables the base detection model to evolve in response to environmental shifts without reliance on original datasets, achieving enhancement through ongoing adjustments during the inference phase. During the test-time adaptation, the model incrementally adapts to incoming target domain data $x_{T}^{\left(t\right)}$ with the CTTA-DisDet framework generating respective adapted models $\theta_{S}^{\left((t)\right)}$ and $\theta_{T}^{\left((t)\right)}$ for each image *t*. This process facilitates more accurate predictions for each subsequent target data point $x_{T}^{\left(t\right)}$.

In agriculture, the environment around crops is constantly evolving due to changes in geographical location, climatic conditions and time. In this context, the model needs to make accurate perceptual decisions in real time and can quickly adapt to these changes. At test time, the input of the model does not see the unseen target domain data $X_{T}$. The target domain is presented one by one in time series order, which means that at any given time point *t*, the model can only access the target domain data at that moment. Based on the target domain data $x_{T}^{\left(t\right)}$, $\theta_{S}$ must make an immediate prediction to get $\theta_{S}^{\left(0\right)}(x^{\left(t\right)})$ and adjust itself at the same time, so as to better cope with the new data $x^{\left(t+1\right)}$ that may appear later.

**Figure 4:**
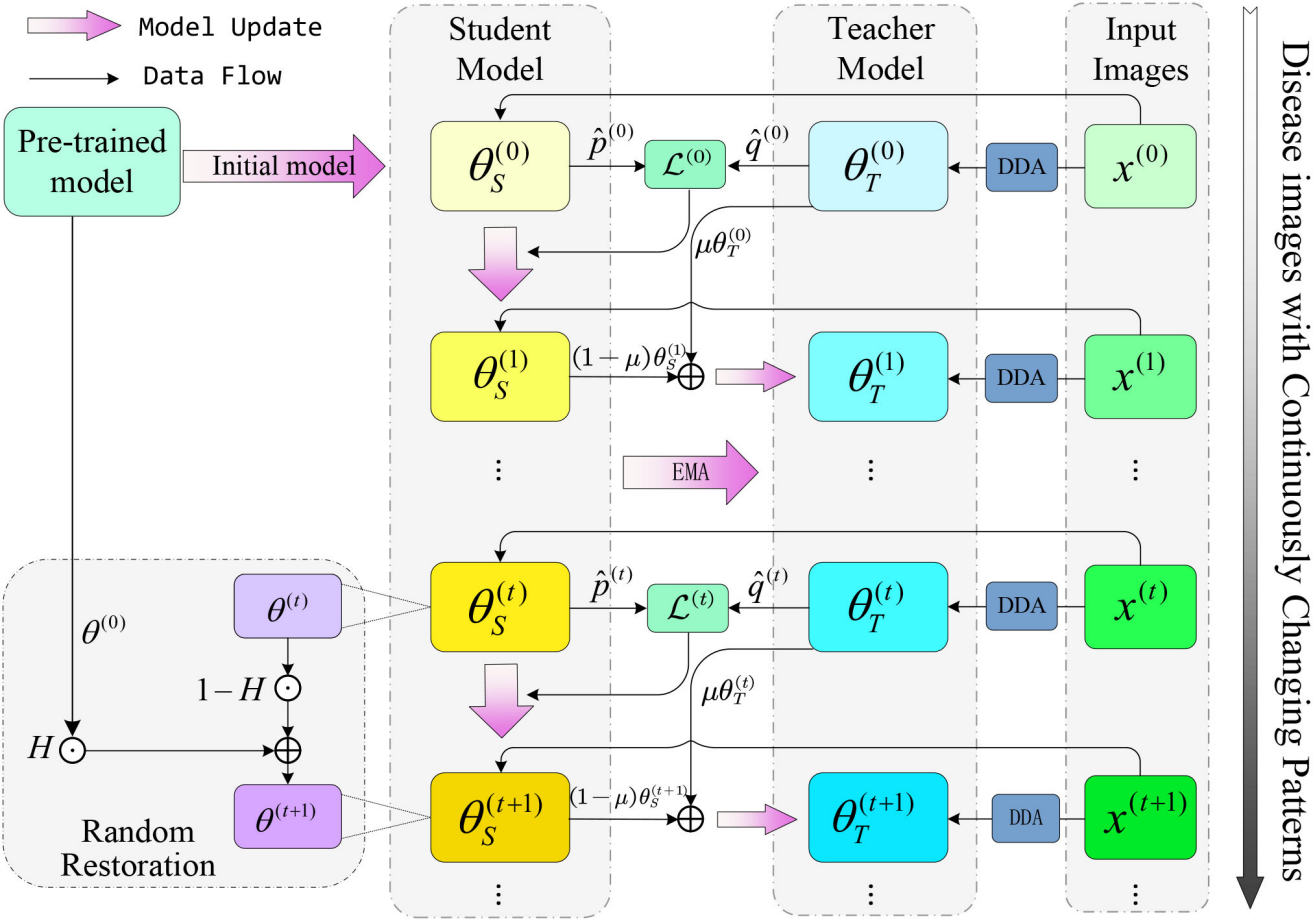
In the continuous test time domain adaptive graph, the teacher model *θ_T_* is used to generate pseudo-labels, and the student model *θ_S_* is used to adapt to the new domain. ⊙ denotes element-by-element multiplication and ⊕ denotes addition.

We introduce an innovative adaptive strategy designed for real-time model adaptability within a fluctuating target domain, as illustrated in **Figure 4**. This strategy leverages a pre-trained model, which is equipped to swiftly respond to domain changes through a dynamic adjustment mechanism. To address the potential issue of error accumulation during self-training, we employ knowledge distillation and pseudo-labeling techniques. For each given test sample ***x****^t^*, the proposed DDA generates explicit and implicit augmented images ***x****exp*^(^*^i^*^)^*i* = 1^16^ and ***x****imp*^(^*^i^*^)^*i* = 1^16^. During testing, 8 images are randomly sampled from these 32 augmentations (denoted as a subset *S*), which are then fed into the teacher model *θ_T_*. The teacher model *θ_T_* generates pseudo-label ${\hat{y}}_{T}^{\left(t\right)}$ as shown in Equation 6:

(6) $${\hat{y}}_{T}^{\left(t\right)}=\frac{1}{8}\sum_{i\in S}\theta_{T}^{\left(t-1\right)}(x_{aug}^{\left(i\right)}),$$

We note that this method modulate pseudo-labels, mitigating error propagation and enhancing the prediction accuracy. Once the pseudo-label is obtained, the student model *θ_S_* is updated by minimizing the cross-entropy loss between the prediction and the pseudo-label to achieve $\theta_{s}^{\left(t\right)}\leftarrow \;\theta_{S}^{\left(t-1\right)}\;$. Then, we utilize the exponential moving average (EMA) to update the teacher model obtain *θ_T_* (Wang et al., 2022; Klinker, 2011). Furthermore, to prevent catastrophic forgetting when the model adapts to new target domains, we incorporate a random restoration mechanism from the base model *θ*^0^. This approach ensures that the model retains essential knowledge from its initial training, integrating this knowledge at strategic points during the adaptation process. Hence, it maintains its foundational expertise while accommodating new domain-specific insights.

Unlike TENT and CoTTA which perform single-step adaptation, CTTA-DisDet introduces a dual-loop mechanism combining EMA-updated teacher and random-restored student for continuous adaptation over time, effectively mitigating catastrophic forgetting and error accumulation. This dynamic frequency adjustment of EMA and stochastic recovery ratio distinguishes our framework from prior TTA approaches.

#### 2.3.1 Continual test-time adaptation

The student model $\theta_{S}^{\left(t\right)}$ refines its predictions by minimizing the loss relative to a teacher model.

The classification loss $L_{cls}^{\left(t\right)}\;$quantifies the categorical discrepancy between the student and teacher model predictions, as defined in Equation 7:

(7) $$L_{cls}^{\left(t\right)}=-\sum_{c}{\hat{p}}_{cls}^{\left(t\right)}\log{\hat{q}}_{cls}^{\left(t\right)},$$

where ${\hat{p}}_{cls}^{\left(t\right)}$ and ${\hat{q}}_{cls}^{\left(t\right)}\;$ represent the teacher and student model’s categorical predictions, respectively.

The confidence loss $L_{conf\;}^{\left(t\right)}$ measures the divergence in confidence scores between the models as shown in Equation 8:

(8) $$L_{conf}^{\left(t\right)}=-\frac{1}{N}\sum_{i=1}^{N}[{\hat{p}}_{conf}^{\left(t\right)}\log{\hat{q}}_{conf}^{\left(t\right)}+(1-{\hat{p}}_{conf}^{\left(t\right)})\text{log}(1-\text{log}{\hat{q}}_{conf}^{\left(t\right)})].$$

Here, *N* denotes the number of prediction boxes, and ${\hat{p}}_{conf}^{\left(t\right)}\;$ and ${\hat{q}}_{conf}^{\left(t\right)}$ represent the confidence scores of the teacher and student models, respectively.

The bounding box loss $L_{bbox}^{t}$ evaluates the spatial accuracy of the predictions, as shown in Equation 9:

(9) $$L_{bbox}^{\left(t\right)}=\sum_{i=1}^{4}\text{Smoot}\_\text{L}1({\hat{p}}_{bbox}^{\left(t\right)},\;{\hat{q}}_{bbox}^{\left(t\right)}).$$

The total loss L at each time step *t* is a weighted sum of these losses as shown in Equation 10:

(10) $$L=\lambda_{cls}L_{cls}^{t}+\lambda_{conf}L_{conf}^{t}+\lambda_{bbox\;}L_{bbox}^{t},$$

where *λ_cls_* = 0.4, *λ_conf_* = 0.3, and *λ_bbox_* = 0.3 are the balancing parameters.

The student model $\;\theta_{s}^{\left(t\right)}$ is then updated using a single gradient descent step with a learning rate *η* = 0.001, as shown in Equation 11:

(11) $$\theta_{S}^{\left(t\right)}\leftarrow \;\theta_{S}^{\left(t-1\right)}-\eta \cdot \nabla L^{\left(t\right)}.$$

#### 2.3.2 With the adapted student model, the final prediction can be achieved, that is, [*q_cls_, q_conf_, q_bbox_*] = $\theta_{S}^{\left(t\right)}(x^{\left(t\right)}).$Exponential moving average

A key objective in self-training is aligning model predictions with generated pseudo-labels, typically achieved by minimizing the cross-entropy between predictions and pseudo-labels for target data $x_{T}^{\left(t\right)}$ and model $\theta_{S}^{\left(t\right)}$. While using model predictions as pseudo-labels is effective in static domains, it poses challenges in dynamic domains due to potential shifts in data distribution that can degrade pseudo-label accuracy. To address this, we’ve adopted an innovative approach to produce high-quality pseudo-labels. We introduce an exponential moving average (EMA) (Wang et al., 2022; Klinker, 2011) to enhance the model’s adaptability in a continuously evolving target domain. Initially, the teacher model’s weights $\theta_{T}^{\left(t\right)}$ mirror the source pre-trained network. At each time step *t*, the teacher model and student model collaboratively generate the primary prediction ${\hat{q}}^{\left(0\right)}$ and the pseudo-label ${\hat{p}}^{\left(t\right)}$. The teacher model’s weights are updated using EMA, blending with the student model’s weights, with a smoothing factor of *µ* = 0.95 ensuring a smooth transition, as shown in Equation 12:

(12) $$\theta_{T}^{\left(t\right)}=\mu \theta_{T}^{\left(t-1\right)}+(1-\mu )\theta_{S}^{\left(t\right)}.$$

After the teacher model is updated and the student model is fine-tuned, the model is ready to accept the next target domain data point $x_{T}^{\left(t-1\right)}$. This process continues iteratively, with the model adapting to each new data point in real time, ensuring accurate predictions and robust performance in dynamic environments.

#### 2.3.3 Random restoration

While pseudo-labels are crucial for maintaining model accuracy across domains, prolonged self-training can introduce errors, especially in the presence of significant domain shifts. Such distribution changes may result in increasingly inaccurate predictions, potentially leading to a scenario where self-training reinforces false predictions with erroneous labels. Additionally, models may suffer from catastrophic forgetting after adapting to new domains, compromising their performance on the original data. To address it, we introduce a random restoration mechanism (Cui et al., 2023) designed to re-integrate knowledge from the source pre-trained model *θ*^(0)^. Specifically, at the beginning of each iteration of the student model $\theta_{S}^{\left(t\right)}$ the restoration is governed by Equations 13 and 14, it’s parameters will first retained 1 − *p* proportion, and *p* proportion of the parameters will be randomly restored to the base model *θ*^(0)^. This random restoration mechanism ensures that the model retains essential knowledge from the source model during self-training, enhancing its adaptability to new data while preserving its understanding of the original data and preventing catastrophic forgetting due to over-adaptation. Formally, it can be expressed as:

(13) H ∼Bernoulli(p),

(14) $$\theta^{\left(t+1\right)}\;\leftarrow H\;⊙\theta^{\left(0\right)}+(1-H)⊙\theta^{\left(t\right)}.$$

Here, ⊙ denotes element-wise multiplication, *p* is a small restoration probability, and ***H*** is a binary mask. we note that ***H*** determines which weight elements revert to the source weight *θ*(0), allowing us to deliberately recover lost knowledge from the source model and enhance the model’s adaptability to new data without forgetting the original.

The final model prediction leverages the student model’s output, integrating information from past iterations to mitigate performance degradation due to catastrophic forgetting. This approach not only bolsters the model’s robustness during continuous adaptation but also reduces error accumulation from distribution shifts, thereby improving the model’s generalization to new, unseen domains. It ensures not only immediate performance in the target domain but also long-term stability in a dynamically changing environment.

## 3 Experiments

### 3.1 Experiment setting and dataset

Our experiment is based on PyTorch 2.2.2 + Cuda_121 and is trained using NVIDIA GeForce RTX 4090. The random restoration probability *p* = 0.1, the learning rate of student model update is *η* = 0.001, and the smoothing factor in EMA is *µ* = 0.95. Batch size is 16, the number of epoch is 100, and the optimizer is Adam to obtain the base model, where the network architecture is YOLOv9s.

Our dataset uses a self-collected tomato disease dataset, including a training set of 4059, the validation set of 451 and test set of 450. The categories are tomato_healthy, tomato_leaf blight, tomato_leaf curl, tomato_septoria leaf spot and tomato_verticulium wilt. The test set is extended the original image to four fixed explicit including Gaussian noise, Brightness adjustment, Pixel loss and Motion blur, for flexible implicit including Foggy day, Dark night, Rainy day, Snowy day on the basis of the original image to detect the recognition ability of the model in the cross-domain case.

To ensure the reliability of the experiments, we also conducted additional experiments using the PlantDoc public dataset and augmented it into the eight types of cross-domain images mentioned above to evaluate the model’s performance.

### 3.2 Analysis of dynamic data augmentation

Our explicit augmenter utilizes the four fixed augmentation methods, Gaussian noise, brightness adjustment, pixel loss and motion blur to generate 16 augmented images for each input image. Despite the diversity of these fixed augmentations, other parameter-free augmentation strategies may be feasible. To explore this possibility, we introduce two new augmentation method, cropping, rotation, to compare its performance with the four fixed augmentations. Concretely, for the cropping method, we randomly crop 20% square area of the original image, while for the rotation method, we rotate the image by 90 degrees. The experimental results are shown in **Table 1**, where the proposed $A_{exp}$ outperforms the cropping and rotation methods in terms of precision, recall, mAP50, and mAP50-95. Therefore, we can draw the conclusion that although these four explicit augmentation methods each have certain characteristics. Compared with adding one more augmentation method, their influence on the overall experimental results is relatively small. Consequently, it can be concluded that the our four explicit augmentation methods possess representativeness and universality.

**Table 1:** Experimental results of our explicit augmentation methods (Gaussian noise, Brightness adjustment, Pixel loss, and Motion blur), and the combination with other specific augmentation methods (Cropping, Rotation), illustrating the performance of the YOLOv9s model on our custom- built dataset.

Fixed explicit	Precision↑	Recall↑	mAP50↑	mAP50-95↑
The proposed $A_{exp}$	74.5	75.9	81.3	63.9
$A_{exp}$ + Cropping	74.8	75.8	81.0	63.7
$A_{exp}$ + Rotation	74.6	74.9	80.6	63.1

The implicit augmenter $A_{imp}$ generates 16 augmented images for each input image using the four flexible augmentation methods, foggy day, dark night, rainy day, and snowy day. To explore the potential of other flexible augmentation methods, we introduce a new augmentation method, frost day, to compare its performance with the four flexible augmentations. The prompt template is similar to our proposed ones, i.e., Generate different levels frost effect to the given image, and other setting are the same as the four $A_{imp}$ augmentations. As shown in **Table 2**, the proposed $A_{imp}$ clearly outperforms the frost day method in terms of precision, recall, mAP50, and mAP50-95. This indicates that the our proposed four flexible augmentation methods are more effective than the frost day method, where other more complex generation strategies are not necessary.

**Table 2:** Experimental results of our implicit augmentation methods (Foggy day, Dark night, Rainy day, Snowy day), and the combination with another specific augmentation method (Frost day), illustrating the performance of the YOLOv9s model on our custom-built dataset.

Flexible implicit	Precision↑	Recall↑	mAP50↑	mAP50-95↑
The proposed $A_{imp}$	76.0	75.5	81.7	64.9
$A_{imp}$ + Frost	75.7	75.7	81.3	64.7

Analysis of **Table 3** reveals that Dynamic Data Augmentation (DDA) significantly enhances performance metrics—Precision, Recall, mAP50, and mAP50-95—across all models compared to Non-Augmentation and Traditional Augmentation conditions. For YOLOv9c, under Non- Augmentation, the metrics are Precision=63.2%, Recall=70.5%, mAP50 = 69.6%, and mAP50- 95 = 55.0%; Traditional Augmentation improves these to 74.2%, 75.9%, 81.1%, and 64.3%, respectively; while Dynamic Data Augmentation further elevates them to 77.1%, 75.4%, 83.8%, and 65.9%, reflecting gains of 13.9%, 4.9%, 14.2%, and 10.9% over Non-Augmentation, and notable improvements over Traditional Augmentation. Similarly, for other models like YOLOv8s, Dynamic Data Augmentation boosts Precision from 61.5% (Non-Augmentation) and 66.9% (Traditional Augmentation) to 71.5%, and mAP50–95 from 57.5% and 59.5% to 63.7%; for YOLOv8m, mAP50–95 reaches 65.8% under Dynamic Data Augmentation, a 8.6% increase over Non-Augmentation (57.2%). This consistent performance uplift across models underscores the reliability and effectiveness of Dynamic Data Augmentation in enhancing robustness and generalization capability.

**Table 3:** Comparison of various models under non-augmentation, traditional augmentation, and our DDA conditions, illustrate the performance of the models on our custom-built dataset.

Model	Non-augmentation				Traditional aug.				DDA			
	P*↑*	R*↑*	mAP50*↑*	mAP50-95*↑*	P*↑*	R*↑*	mAP50*↑*	mAP50-95*↑*	P*↑*	R*↑*	mAP50*↑*	mAP50-95*↑*
YOLOv8s	61.5	74.9	73.4	57.5	66.9	74.9	75.9	59.5	71.5	76.1	81.4	63.7
YOLOv8m	69.2	68.1	72.4	57.2	73.0	73.9	79.6	62.3	75.8	78.8	83.5	65.8
YOLOv8l	62.9	69.2	66.2	52.3	77.7	72.1	79.9	63.3	79.3	75.9	83.3	65.3
YOLOv9s	61.8	68.9	67.5	53.7	72.6	74.1	79.8	62.9	75.5	73.8	82.2	64.4
YOLOv9c	63.2	70.5	69.6	55.0	74.2	75.9	81.1	64.3	77.1	75.4	83.8	65.9
YOLOv9e	66.3	67.0	67.3	52.6	72.9	74.3	76.6	61.2	73.3	76.6	82.8	65.3
YOLOv10n	59.6	69.1	66.2	50.8	71.1	70.6	75.9	59.2	75.2	68.5	79.6	60.7
YOLO-FMDI	64.9	69.0	69.3	53.1	73.9	74.1	79.8	63.5	74.6	75.4	82.8	65.7
Faster-RCNN	42.7	47.3	49.5	37.2	61.3	58.2	63.6	45.1	64.5	61.1	67.7	48.2

Metrics include Precision (P), Recall (R), Mean Average Precision at IoU=0.5 (mAP50), and Mean Average Precision from IoU=0.5 to 0.95 (mAP50-95)(/%).

Additionally, we employed the DDA algorithm to test image augmentation under a single scenario, with the results presented in **Table 4**. By comparing the experimental outcomes, we observed that while image augmentation in a single scenario may not be as effective as composite augmentation, it still outperforms traditional methods of image augmentation. It is noteworthy that although the four distinct augmentation scenarios each demonstrated certain advantages when used individually, the performance differences among them were not significant.

**Table 4:** Comparison of various models under DDA with different single-scenario augmentations, illustrate the performance of the models on our custom-built dataset.

Model	DDA (foggy day)				DDA (dark night)				DDA (rainy day)				DDA (snowy day)			
	P↑	R↑	mAP50↑	mAP50-95↑	P↑	R↑	mAP50↑	mAP50-95↑	P↑	R↑	mAP50↑	mAP50-95↑	P↑	R↑	mAP50↑	mAP50-95↑
YOLOv8s	69.5	74.1	78.8	61.7	70.2	74.6	79.1	62.1	69.8	74.8	79.4	62.3	70.0	74.3	78.9	61.9
YOLOv8m	73.3	76.5	81.8	63.7	74.1	77.0	82.2	64.1	74.4	77.3	82.4	64.4	73.8	76.8	82.1	63.8
YOLOv8l	77.5	73.9	81.5	63.3	77.1	74.3	81.9	63.5	77.8	74.7	82.1	63.9	77.2	74.5	81.7	63.2
YOLOv9s	74.8	73.0	79.8	63.6	75.4	73.4	79.9	64.1	75.4	73.8	80.5	64.0	74.5	73.2	79.6	63.5
YOLOv9c	75.4	73.5	81.2	64.1	75.8	74.0	81.5	64.5	75.9	74.2	81.5	64.6	75.2	73.7	81.3	64.0
YOLOv9e	71.1	75.0	79.8	63.3	71.7	75.4	80.2	63.7	71.9	75.8	80.4	63.9	71.5	75.3	79.9	63.1
YOLOv10n	73.1	66.8	76.8	59.3	73.4	67.2	77.1	59.6	73.7	67.0	77.3	59.9	73.3	66.6	76.7	59.5
YOLO-FMDI	72.5	73.2	79.8	63.7	73.2	73.8	80.1	64.1	73.0	74.0	80.4	64.5	72.8	73.6	80.0	64.0
Faster-RCNN	62.1	59.3	64.7	46.8	62.9	59.8	65.3	47.1	62.7	59.4	65.9	47.4	62.5	59.9	65.0	46.9

a Metrics include Precision (P), Recall (R), Mean Average Precision at IoU=0.5 (mAP50), and Mean Average Precision from IoU=0.5 to 0.95 (mAP50-95)(/%).

**Figure 5:**
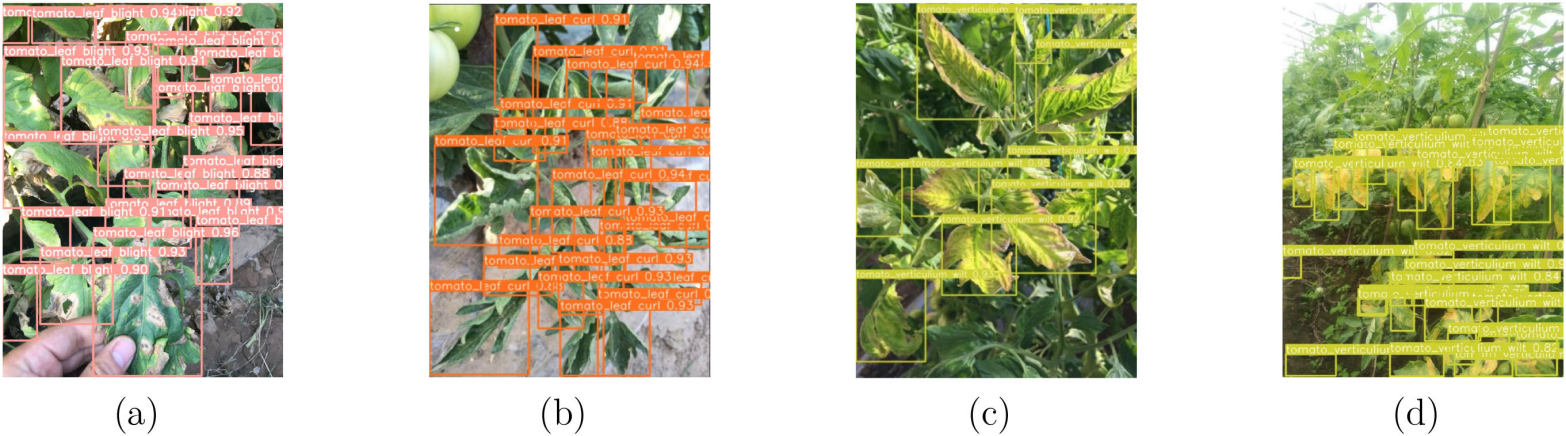
The detection results of the target domain adaptation model in the source domain are illustrated. **(a)** tomato leaf blight, **(b)** tomato leaf curl, **(c)** tomato verticillium wilt, and **(d)** tomato verticillium wilt.

**Table 4** further examines the effect of each single-scenario augmentation within DDA, providing granular evidence of how different weather conditions influence cross-domain robustness; thus it complements **Table 3**; **Figure 5** rather than duplicating them.

Image augmentation in a single scenario, though less comprehensive than composite augmentation, can still enhance performance. This is significant for applications with limited resources or specific needs, showing that simple augmentation can be beneficial without complex strategies.

Furthermore, this insight also indicates that when devising image augmentation strategies, there is no need to overly pursue complex composite methods. Instead, the most appropriate augmentation strategy should be selected based on the actual application scenario and available resources. In some cases, a simple and effective single-scenario augmentation may be sufficient to meet the requirements, thereby conserving computational resources and time.

**Figure 6:**
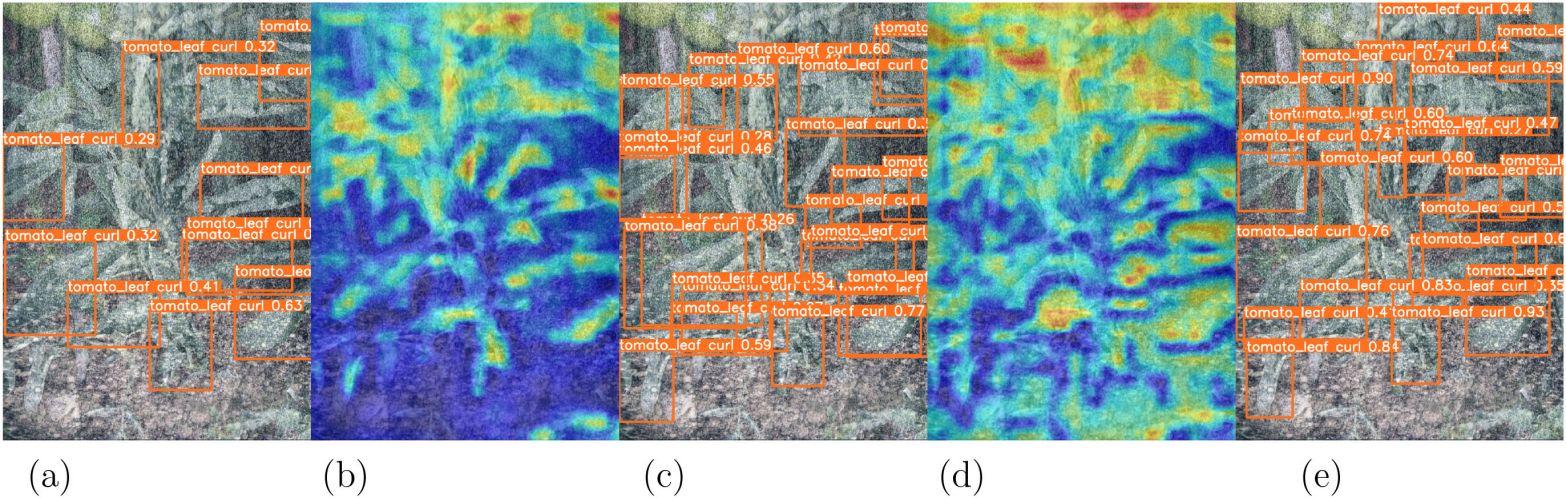
Comparison of the baseline model, the traditional augmentation and DDA (original diagram and heat map). **(a)** YOLOv9c, **(b)** Heat map of baseline, **(c)** Traditional Augmentation, **(d)** Heat map of Traditional Augmentation, and **(e)** DDA.

It can be seen from **Figure 6** that the confidence of the model trained after adding the DDA to the detection target is significantly increased, with an average increase of 0.175, and the attention distribution map is more focused on visualizing plant disease sites. This shows that the DDA significantly improves the cross-domain recognition ability of the model.

**Figure 7:**
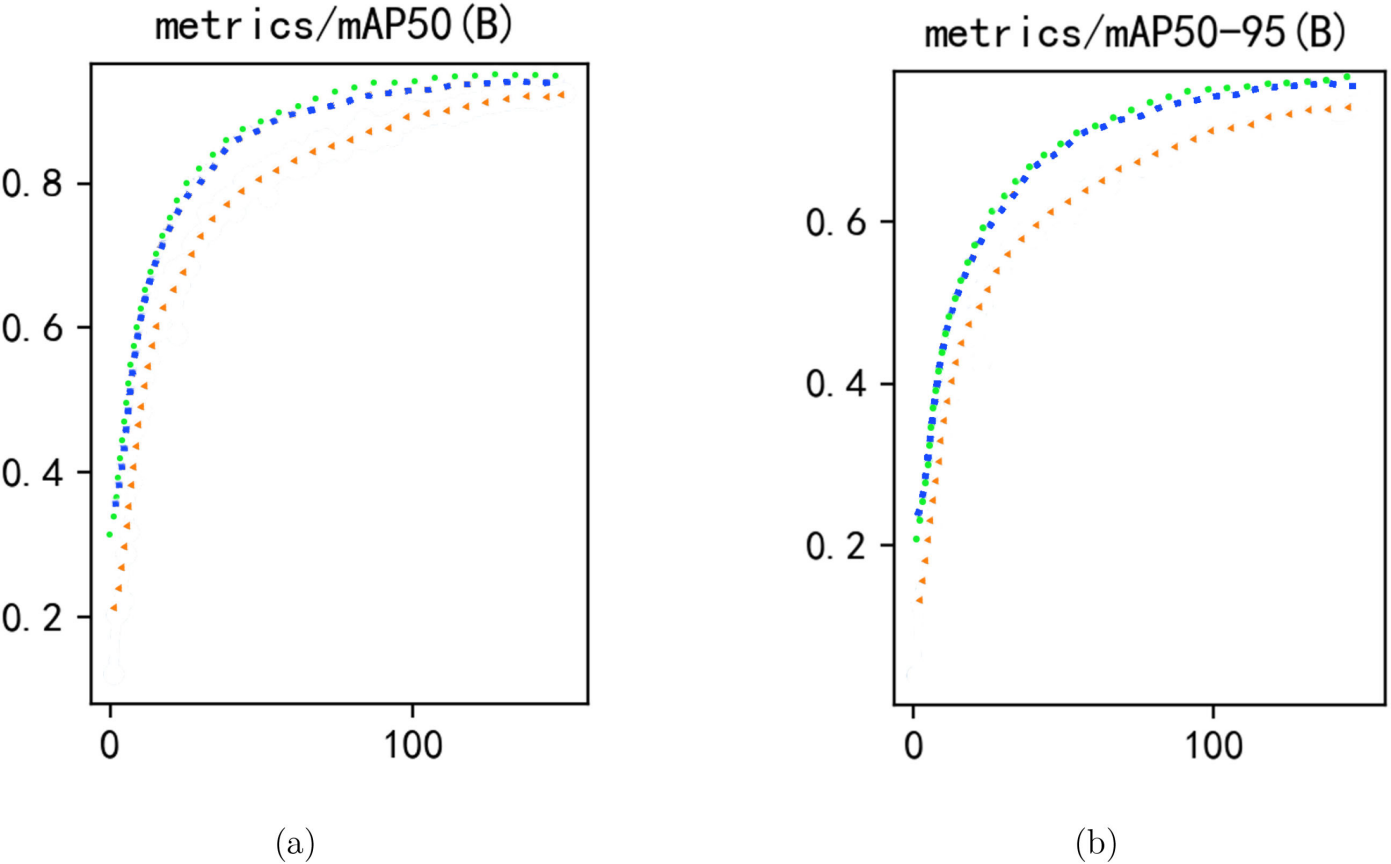
Comparison of non-augmentation (orange triangles), traditional augmentation (blue squares), and DDA (green circles). **(a)** metrics/mAP50 (B) and **(b)** metrics/mAP50–95 (B).

In the diagram **Figure 7**, orange(triangle) is the source model, blue(rectangle) is the traditional enhancement and green(circular) is DDA. It can be clearly seen that in the data of the source domain, the model learning of DDA is faster and the same accuracy is achieved in the earlier time and the final accuracy is higher. It can be seen that the final accuracy DDA *>* Traditional enhancements *>* Source model.

### 3.3 Comparison of cross-domain detection performance

Analyzing **Table 5**, this table comprehensively presents the performance of various methods on a custom tomato dataset under two conditions—Traditional Augmentation and DDA—across different augmentation types, with performance measured by mAP50. A detailed examination of the data reveals the outstanding effectiveness and robustness of CTTA-DisDet(v9s) in handling varying environmental conditions under both augmentation scenarios. Under Traditional Augmentation, CTTA-DisDet(v9s) achieves an average mAP50 of 65.3%, surpassing other methods such as YOLOv9s (60.6%), BN (57.2%), PL (36.3%), TENT (35.5%), LAME (63.3%), and CoTTA (62.1%), with particularly strong results in specific augmentations like FO (49.4%), PL (71.5%), DA (76.5%), GN (77.4%), BR (72.7%), and MB (58.9%), where it consistently secures the highest or near-highest scores, highlighting its superior adaptability across diverse image distortion scenarios. When evaluated under DDA, CTTA-DisDet(v9s) further improves its average mAP50 to 67.9%, again outperforming YOLOv9s (63.1%), BN (60.3%), PL (39.1%), TENT (38.5%), LAME (66.4%), and CoTTA (64.8%), while maintaining a lead in augmentations such as FO (51.5%), SN (53.4%), PL (73.8%), DA (79.1%), GN (80.1%), BR (74.9%), and MB (62.2%), even surpassing methods like LAME (e.g., SN: 64.4%) and CoTTA (e.g., GN: 79.2%) that excel in specific categories. Notably, CTTA-DisDet(v9s) demonstrates consistent improvement from Traditional Augmentation to DDA, with gains such as FO increasing from 49.4% to 51.5%, PL from 71.5% to 73.8%, and GN from 77.4% to 80.1%, underscoring how DDA enhances the model’s resilience to complex environmental variations. Overall, CTTA-DisDet(v9s) not only achieves a significantly higher average performance compared to other methods but also excels in most individual augmentation types, validating its effectiveness in leveraging dynamic data augmentation and continuous test-time domain adaptation to boost detection accuracy and robustness in non-stationary agricultural settings like tomato disease detection, thereby offering reliable support for real-world applications.

**Table 5:** Performance of various methods on the custom tomato dataset under different augmentations (mAP50/%).

Time	⎯⎯⎯⎯⎯⎯⎯⎯⎯⎯⎯⎯⎯⎯⎯⎯⎯⎯⎯⎯⎯⎯⎯⎯⎯⎯⎯⎯⎯⎯⎯⎯⎯⎯⎯⎯⎯⎯⎯⎯⎯⎯⎯⎯⎯⎯⎯⎯⎯⎯⎯⎯⎯⎯⎯⎯→								
Traditional aug.	FO	RA	SN	PL	DA	GN	BR	MB	Avg
YOLOv9s	44.2	65.3	39.1	63.2	76.3	73.4	68.1	55.2	60.6
BN	44.7	65.1	47.3	56.5	63.4	59.0	66.0	55.4	57.2
PL	40.0	33.2	25.0	36.0	38.0	43.2	51.3	23.3	36.3
TENT	37.3	34.5	30.1	40.6	33.3	43.2	44.2	21.0	35.5
LAME	41.9	64.2	61.8	71.3	79.2	63.3	67.0	57.8	63.3
CoTTA	45.1	66.6	44.2	64.1	73.2	77.1	70.2	56.6	62.1
CTTADisDet( v9s)	49.4	65.2	51.1	71.5	76.5	77.4	72.7	58.9	62.3
DDA	FO	RA	SN	PL	DA	GN	BR	MB	Avg
YOLOv9s	46.3	68.2	41.2	66.3	78.2	76.3	70.1	58.3	63.1
BN	48.5	67.3	51.2	59.8	67.4	60.9	69.6	57.3	60.3
PL	42.5	35.9	27.5	39.0	40.9	46.9	53.9	26.6	39.1
TENT	40.3	37.3	33.0	43.1	36.9	45.8	47.6	23.7	38.5
LAME	44.6	67.5	64.4	74.7	81.9	67.4	70.0	60.9	66.4
CoTTA	47.2	68.1	47.1	67.2	77.1	79.2	73.1	59.2	64.8
CTTADisDet( v9s)	51.5	68.1	53.4	73.8	79.1	80.1	74.9	62.2	67.9

Augmentations: FO, Foggy day; RA, Rainy day; SN, Snowy day; PL, Pixel loss; DA, Dark night; GN, Gaussian noise; BR, Brightness adjustment; MB, Motion blur.

Analyzing **Table 6**, this table evaluates adaptation performance on the PlantDoc dataset under diverse environmental augmentations. While YOLOv9s achieves competitive scores in specific traditional augmentation scenarios (e.g., FO: 50.6%, PL: 51.4%, DA: 56.7%), its performance fluctuates significantly across conditions like SN (27.1%) and MB (20.5%), exposing vulnerability to extreme distortions. CTTA-DisDet(v9s) demonstrates balanced robustness, attaining the highest average mAP50 in both Traditional Aug. (46.7%) and DDA (49.8%) without extreme performance drops. Notably, it addresses critical weaknesses of specialized methods: under DDA, CTTA-DisDet(v9s) surpasses LAME’s strong MB performance (43.1% vs. 42.9%) while outperforming CoTTA in SN (42.6% vs. 43.4%) and GN (49.3% vs. 38.7%), showcasing multi- threat mitigation capability. The method exhibits strategic improvements from Traditional Aug. to DDA, particularly enhancing RA (45.3%→48.4%) and GN (46.2%→49.3%), where other approaches plateau. Unlike the tomato dataset analysis, PlantDoc reveals CTTA-DisDet(v9s)’s ability to narrow the performance gap between specialized augmentations (e.g., improving RA by 3.1% while maintaining FO gains), achieving more homogeneous robustness across all test conditions. This contrasts with LAME’s inconsistent adaptation, which excels in MB (43.1%) but falters in FO (43.1%). The results validate CTTA-DisDet(v9s)’s cross-dataset effectiveness, particularly in handling PlantDoc’s complex multi-class detection scenarios through stable, non-oscillating adaptation.

**Table 6:** Performance of various methods on the PlantDoc dataset under different augmentations (mAP50/%).

Time	⎯⎯⎯⎯⎯⎯⎯⎯⎯⎯⎯⎯⎯⎯⎯⎯⎯⎯⎯⎯⎯⎯⎯⎯⎯⎯⎯⎯⎯⎯⎯⎯⎯⎯⎯⎯⎯⎯⎯⎯⎯⎯⎯⎯⎯⎯⎯⎯⎯⎯⎯⎯⎯⎯⎯⎯→								
Traditional aug.	FO	RA	SN	PL	DA	GN	BR	MB	Avg
YOLOv9s	50.6	40.3	27.1	51.4	56.7	41.5	48.7	20.5	42.1
BN	45.8	43.4	33.3	45.2	49.9	35.7	45.2	26.2	40.6
PL	43.2	31.7	24.9	38.1	44.6	33.1	38.1	12.0	33.2
TENT	39.5	36.2	27.6	37.4	39.6	33.8	36.8	12.1	32.9
LAME	40.4	42.4	41.3	49.9	52.9	42.1	49.3	39.7	44.7
CoTTA	42.5	43.6	42.1	45.7	54.8	36.0	45.5	29.6	42.5
CTTADisDet (v9s)	47.4	45.3	39.5	49.3	54.5	46.2	51.3	39.8	46.7
DDA	FO	RA	SN	PL	DA	GN	BR	MB	Avg
YOLOv9s	53.6	43.8	29.9	54.7	59.4	44.9	52.3	24.1	45.3
BN	48.4	46.4	36.0	48.5	52.3	38.8	48.0	29.4	43.5
PL	45.9	34.1	27.8	40.3	47.4	35.6	41.1	14.3	35.8
TENT	42.6	38.7	30.4	39.8	42.5	36.4	39.8	14.4	35.6
LAME	43.1	48.9	40.1	49.2	60.5	46.2	49.2	43.1	47.5
CoTTA	45.7	46.2	43.4	45.6	58.2	38.7	48.6	32.4	44.9
CTTADisDet (v9s)	50.5	48.4	42.6	52.4	57.6	49.3	54.4	42.9	49.8

Augmentations: FO, Foggy day; RA, Rainy day; SN, Snowy day; PL, Pixel loss; DA, Dark night; GN, Gaussian noise; BR, Brightness adjustment; MB, Motion blur.

To demonstrate that the model does not experience catastrophic forgetting while enhancing detection capability in the target domain and retaining the knowledge acquired from the source domain, we conducted a re-evaluation on the source domain after the model adapted to the target domain. As shown in **Figure 5**, the accuracy of the model remains consistent with that of the version without cross-domain adaptation, indicating that our method of random recovery effectively prevents catastrophic forgetting.

**Figure 8:**
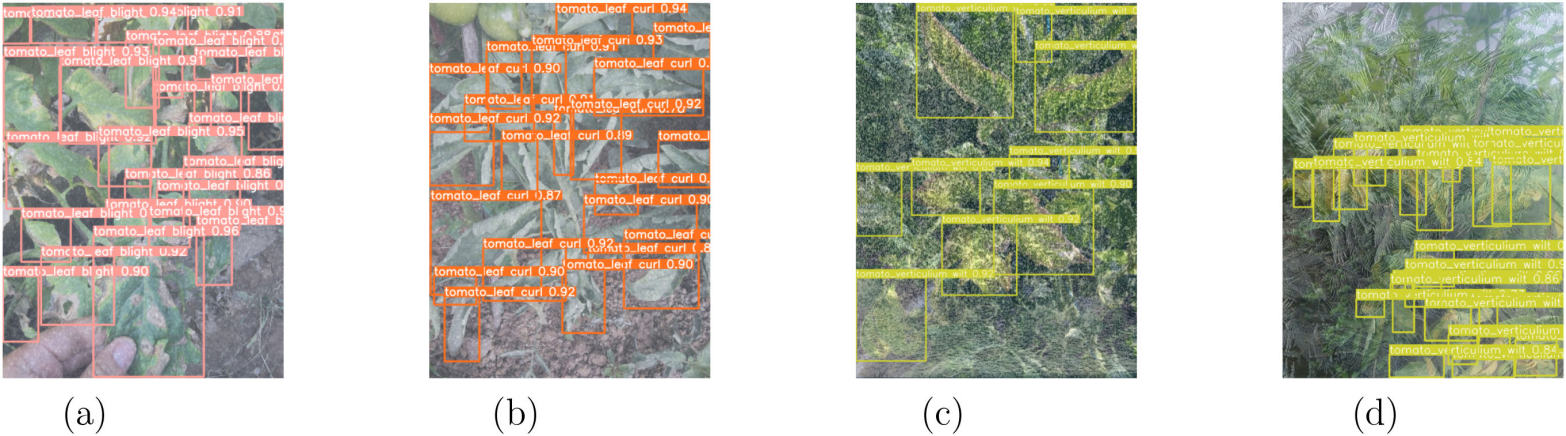
CTTA-DisDet experimental results diagram under different scenarios. **(a)** Snowy, **(b)** Foggy, **(c)** Rainy, and **(d)** Frost.

Analyzing **Table 7**, CTTA-DisDet’s FPS of 47 is notably lower than YOLOv9s (163) and YOLOv9e (55), which is closely tied to its significantly higher computational load of 303.64 GFLOPs compared to 26.9 for YOLOv9s and 189.5 for YOLOv9e; however, its precision (Avg. mAP50) reaches 67.9, substantially surpassing YOLOv9s (60.6) and YOLOv9e (60.2), highlighting its superior accuracy and reliability in object detection tasks; in terms of parameters, CTTA-DisDet (14,636,736) falls between the two, suggesting that its complexity is not solely driven by parameter scale but rather by computational intensity; although its FPS is lower, 47 frames per second remains sufficient for real-time processing in many practical applications, and it even offers advantages in precision-critical scenarios; thus, despite its high computational demand, CTTA-DisDet effectively meets usage requirements through its enhanced precision, striking a reasonable balance between performance and practicality. Furthermore, as shown in **Figure 8**, the experimental results of CTTA-DisDet under different scenarios demonstrate the model’s robust detection capability and adaptability across diverse field conditions.

**Table 7:** Evaluation of methods’ efficiency.

Method	Parameters	GFLOPs	FPS	Avg. mAP50
YOLOv9s	7,318,368	26.9	163	60.6
YOLOv9e	58,206,592	189.5	55	60.2
CTTA-DisDet	14,636,736	303.64	47	67.9

## 4 Discussion

In this study, by proposing an innovative framework, through dynamic data augmentation, the model can learn more diverse feature representations, so as to achieve more accurate pest identification in unseen fields. The continuous test time domain adaptive approach allows the model to adjust in real time in the changing target domain, maintaining a rapid response to new situations. Our framework of CTTA-DisDet leverages a teacher-student model configuration, where both models share the same architecture. The teacher model is trained on augmented data, gaining generalized knowledge, which it then transfers to the student model through knowledge distillation. This setup allows the student model to be fine-tuned for specific target domains, significantly enhancing its detection accuracy across different environmental contexts. These methods not only enhance the generalization ability of the model, but also improve its cross-domain detection performance, significantly improve the model’s ability to detect crop diseases in unseen fields, which has important practical significance for improving crop yield and quality. However, it is important to note that CTTA-DisDet may demand higher computational resources which could limit the model’s application in resource-constrained environments. Moreover, while a random recovery mechanism has been implemented to address catastrophic forgetting, further research is needed to ensure the model’s stability and efficiency during long-term adaptation.

## 5 Discussion

In this study, by proposing an innovative framework, through dynamic data augmentation, the model can learn more diverse feature representations, so as to achieve more accurate pest identification in unseen fields. The continuous test-time domain adaptive approach allows the model to adjust in real time in the changing target domain, maintaining a rapid response to new situations. Our framework of CTTA-DisDet leverages a teacher–student model configuration, where both models share the same architecture. The teacher model is trained on augmented data, gaining generalized knowledge, which it then transfers to the student model through knowledge distillation.

This setup allows the student model to be fine-tuned for specific target domains, significantly enhancing its detection accuracy across different environmental contexts. These methods not only enhance the generalization ability of the model, but also improve its cross-domain detection performance, significantly improving the model’s ability to detect crop diseases in unseen fields, which has important practical significance for improving crop yield and quality.

From the experimental results, it can be observed that the integration of dynamic data augmentation (DDA) and continuous test-time adaptation (CTTA) leads to consistent improvements in precision, recall, and mAP across multiple datasets. The explicit augmentations contribute to robustness against common environmental distortions, while the implicit augmentations generated by large language models introduce realistic weather variations that effectively simulate non-stationary conditions. This demonstrates that the proposed dual-augmentation strategy can successfully narrow the domain gap between training and testing data, enabling stable adaptation even under rapid environmental shifts. Furthermore, the dual-loop mechanism combining exponential moving average (EMA) updates with stochastic restoration effectively mitigates catastrophic forgetting, maintaining model stability during long- term adaptation.

Beyond empirical performance, the broader implication of CTTA-DisDet lies in its potential for real-world agricultural automation. By enabling models to self-adapt without source data access, this framework provides a practical pathway for intelligent monitoring systems capable of long-term deployment in complex, evolving environments. However, it is important to note that CTTA-DisDet may demand higher computational resources, which could limit its application in resource-constrained edge devices. Additionally, the fidelity of implicit augmentations still depends on the generative quality of LLM-based image synthesis, which may vary across domains. Future research should explore lightweight adaptation strategies, uncertainty-aware pseudo-label filtering, and hybrid augmentation mechanisms to further enhance stability and efficiency during continuous adaptation.

## Data Availability

The required data has been deposited in a public repository. It is available on Zenodo at the following https://doi.org/10.5281/zenodo.17659449.
